# Impact of physiotherapy with telerehabilitation on caregivers of patients with neurological disorders: A systematic review protocol

**DOI:** 10.3389/fnagi.2022.951397

**Published:** 2022-09-02

**Authors:** Emília Márcia Gomes de Souza e Silva, Luciana Protásio de Melo, Aline Alves de Souza, Ledycnarf Januário de Holanda, Tatiana Souza Ribeiro, Ricardo Alexsandro de Medeiros Valentim, Ana Raquel Lindquist

**Affiliations:** ^1^Laboratory of Intervention and Analysis of Movement, Department of Physical Therapy, Federal University of Rio Grande do Norte, Natal, Brazil; ^2^Laboratory of Technological Innovation in Health, Federal University of Rio Grande do Norte, Natal, Brazil; ^3^Department of Biomedical Engineering, Federal University of Rio Grande do Norte, Natal, Brazil

**Keywords:** caregiver, physical therapy specialty, nervous system diseases, caregiver burden, telemedicine

## Abstract

**Introduction:**

Caregivers are essential during and after rehabilitation but exhibit intense physical and mental burdens due to responsibilities, resulting in stress, irritability, depression, anxiety, pain, and financial distress. Telerehabilitation offers several remote health services that improve time, engagement, and physical and mental health care access. Thus, we outlined a systematic review protocol to evaluate the impact of telerehabilitation on the burden, stress, pain, and quality of life of caregivers of patients with neurological disorders.

**Methods:**

Searches will be conducted in Ovid MEDLINE, Pubmed, Scopus, Web of Science, Embase, Cochrane Central Register of Controlled Trials, Physiotherapy Evidence Database, and PsycINFO databases. Clinical trials evaluating the burden, stress, pain, and quality of life of caregivers of patients with neurological disorders using telerehabilitation will be included without publication date or language restriction. Two reviewers will independently select studies from titles, abstracts, and reference lists. The quality of evidence and risk of bias will be assessed according to Cochrane recommendations.

**Results:**

This systematic review to be developed will evaluate the impact of telerehabilitation on the burden, stress, pain, and quality of life of caregivers of patients with neurological disorders.

**Discussion:**

Caregivers, especially of patients with neurological disorders, need more attention since the overload, stress, duties with other personal responsibilities, and low remuneration may impact the quality of life. Therefore, they need intervention, especially physical therapy via telehealth, which values the time of caregivers and may change their perception of health and quality of life.

**PROSPERO registration number:**

CRD42022278523.

## 1. Introduction

According to the World Health Organization, life expectancy and chronic diseases (especially neurological disorders) increased exponentially in the last decade. Patients with neurological disorders often isolate themselves, stay long periods at home, and require care and attention from formal or informal caregivers (Ploeg et al., 2017; Dixe et al., 2019). Formal caregivers are professionals paid to take care of patients that need help in daily activities. The impact of the caring activity on quality of life and burden of formal caregivers differs from informal caregivers. In contrast, formal caregivers are paid and trained to meet the demands of patients and experience lower overload and stress than informal caregivers (Dixe et al., 2019).

Informal caregivers usually are family members with several responsibilities to help patients with disabilities (e.g., treat wounds, perform hygiene, and control medication, appointments, and therapy schedules), besides personal and professional responsibilities (Ploeg et al., 2017). The burden varies according to the needs of patients (Dixe et al., 2019) and may lead to acute and chronic physical disorders, isolation, depression, financial distress, and self-blame (Abreu Paiva et al., 2019). Moreover, they may present difficult intra-family relationships, economic impairments due to social stigma, and an increased probability of physical suffering (Nogueira et al., 2019). These caregivers are not considered professionals and receive limited training regarding basic care for patients with disabilities; (Chiao et al., 2015) therefore, they may experience intense burden, depression, pain, and low aerobic endurance compared with formal caregivers (Llach et al., 2004; Gusi et al., 2009; Farran et al., 2016).

The physical and psychological benefits of exercise on the daily life of caregivers are well-established (Lambert et al., 2016). For instance, exercise was associated with reduced self-reported stress, chronic diseases, and depressive symptoms and increased self-perception of wellbeing, quality of life, and mobility (Loi et al., 2014; Baik et al., 2021). However, the demands and responsibilities of the caregiver hamper their adherence to health promotion measures (Hearn et al., 2019).

In this context, telerehabilitation emerges as a tool to ensure greater health service efficiency. Telerehabilitation is defined as the delivery of rehabilitation interventions via telecommunication technologies and has been used to optimize time and demonstrate that users can have remote and effective access to physical and mental health, self-care, and physical exercise (Hearn et al., 2019). This strategy also presents several advantages (e.g., low travel costs and flexible schedules) and can be integrated into evaluation processes, health monitoring, and education (Van Egmond et al., 2018).

The health conditions of caregivers of patients with chronic diseases are often precarious due to self-care barriers (e.g., difficulties in displacement, living distant from urban centers, or lack of time; Saragiotto et al., 2020) therefore, new strategies must be developed to increase the access of caregivers to health services.

The acceptability and feasibility of telerehabilitation by patients and their caregivers is already proven in the literature (Geronimo et al., 2017). Furthermore, evidence related to telerehabilitation proves the ability of this type of intervention to reduce characteristics associated with caregiver burden, such as time and financial aspects, since, by carrying out the intervention remotely, the caregiver is able to reduce the time spent traveling and the financial resources consumed (Tindall and Huebner, 2009). In addition, interventions with telerehabilitation aiming at controlling caregiver stress and providing emotional care resulted in a reduction in participants' depressive complaints (Smith et al., 2012).

Moreover, studies using physiotherapy with telerehabilitation either focused on patient rehabilitation or proposed interventions to manage the burden of caregivers (Won et al., 2008; Prick et al., 2011; Forducey et al., 2012; Manceau et al., 2014; Yilmaz et al., 2019). Although well-established, Forducey et al. (2012) and Chen et al. (2017) literature still lacks evidence regarding the impact of physiotherapy with telerehabilitation on health of the caregiver. Thus, we outlined a systematic review protocol to evaluate the impact of telerehabilitation on the burden, stress, pain, and quality of life of caregivers of patients with neurological disorders.

## 2. Methods

### 2.1. Eligibility criteria

Eligibility criteria were established according to the PICOTS strategy (Participants, Interventions, Comparisons, Outcomes, Time frame, and Study design). Clinical trials using telerehabilitation and evaluating the burden, stress, pain, and quality of life of caregivers of patients with neurological disorders will be included with no language or publication date restrictions.

- Types of participants
  We will consider studies using telerehabilitation in formal or informal adult caregivers (>18 years old) of both sexes who took care of patients with any neurological disorder (associated or not with other non-neurological conditions).
- Types of intervention
  Clinical trials addressing the following interventions will be included: (i) caregiver-centered, (ii) patient-centered, (iii) patient-centered and caregiver-mediated.
  Interventions must have been conducted remotely (synchronously or asynchronously). Studies using physiotherapy with telerehabilitation alone or associated with other interventions will be considered. Those not evaluating caregivers will be excluded.
- Types of comparison
  We will include studies comparing the impacts of physiotherapy with telerehabilitation on caregivers of patients with neurological disorders and (1) any type of in-person intervention, either physiotherapy or not; (2) any type of remote intervention other than physiotherapy; and (3) placebo or no intervention.
- Types of outcome measures
  Studies using physiotherapy with telerehabilitation and evaluating any outcomes related to caregivers will be included. However, we will consider the burden of caregivers, analyzed using the Zarit Scale and the Caregiver Burden Inventory, as primary outcome. The following variables will be considered secondary outcomes: stress (Perceived Stress Scale and Lipp Inventory of Stress Symptoms for Adults), quality of life (World Health Organization Quality of Life Brief and Short-Form 36), pain (McGill Pain Questionnaire and short form of the Brief Pain Inventory), and any adverse events reported by caregivers (i.e., falls, hospitalization, or death).
- Time considered for measurement
  Outcome measures will be considered at the following evaluation times:
  1. Before the intervention: the last measurement before the intervention (baseline);
  2. After the intervention (short term): the first measure after the end of the intervention (post-training);
  3. After the intervention (medium or long term): the second measure after the end of the intervention (follow-up).
- Type of studies
  This review will consider only interventional studies, including randomized, quasi−randomized, or non-randomized clinical trials. Crossover studies will be included if they present separate data for each phase. We will exclude case reports, quasi−experimental (before−after) studies, review, letters to the editors, book chapters, and duplicate articles.

### 2.2. Research methods for study selection

#### 2.2.1. Electronic surveys

The search strategy was developed using the Ovid MEDLINE database (from 1946) (Appendix 1) and adapted to the following electronic databases: Pubmed, Scopus, Web of Science, Embase, Cochrane Central Register of Controlled Trials (Cochrane Library; latest issue), Physiotherapy Evidence Database, and PsycINFO. Studies will also be hand-searched from the reference list of included studies.

#### 2.2.2. Search strings

Studies will be selected without language and publication date restrictions. Searches will be refined using terms related to caregivers, telerehabilitation, burden, and quality of life.

#### 2.2.3. Data management

Firstly, two reviewers (EMGSS and AAS) will independently select studies following the eligibility criteria within a random sample of 10% of studies. Once the consensus was identified, the final selection of articles will be performed, according to the screening process summarized in the PRISMA flowchart (Annex I) (Moher et al., 2009). Duplicates will be removed, and any disagreements will be solved by consensus in group sessions.

#### 2.2.4. Data collection

A table will be created for data extraction, and two reviewers will independently evaluate and extract the following information from studies: study design, author(s), and year of publication, and location; clinical diagnosis of people receiving care (injury time); characteristics of caregivers (i.e., gender, age, time of care, relationship with the patient); assessment (i.e., main outcome measurements and scales); intervention (type of intervention, telerehabilitation application, frequency, and duration); and results (Appendix 2).

#### 2.2.5. Dealing with missing data

Researchers will be contacted to verify the main study characteristics and obtain missing outcome data. When not possible or if missing data lead to serious bias, we will conduct a sensitivity analysis to determine the impact of including these studies.

#### 2.2.6. Risk of bias in individual studies

Two reviewers (EMGSS and AAS) will use the Cochrane Risk of bias (RoB) tool, according to the type of study to be analyzed (Higgins and Altman, 2008). Any disagreements will be solved with a third reviewer (ARRL).

The RoB in non-randomized and randomized trials will be assessed using the both Risk of Bias in Non-Randomized Studies (ROBINS-I) and the Cochrane RoB tool for randomized studies (ROB), respectively. The tools are divide bias into seven domains: confounding, selection of participants, classification of interventions, deviations from intended interventions, missing data, measurement of the outcome, and selection of the reported result (Higgins and Altman, 2008).

The RoB assessment will also consider the evaluator blinding. Thus, a high risk of bias will be attributed if participants and evaluators were not blinded, and results could be influenced by participants or knowledge of the team regarding the proposed treatment (Higgins and Altman, 2008).

Studies will be classified as low, high, or unclear (when missing data is identified or the risk of bias is uncertain). If the RoB is unclear due to insufficient information, authors will be contacted to obtain more information and categorize the Ro.

#### 2.2.7. Methodological quality

Quality of evidence will be assessed according to the GRADE system (Guyatt et al., 2011) and classified as high, moderate, low, or very low. Randomized trials without selection bias will be considered for high-quality evidence.

#### 2.2.8. Subgroup analyzes

The following subgroup analyzes will be performed to determine the effectiveness of telerehabilitation on the burden of caregivers of patients with neurological disorders: age, gender, non-progressive vs. progressive neurological disorders, and type of relationship with the patient.

### 2.3. Sensitivity analysis and heterogeneity

Heterogeneity between studies will be assessed using the I^2^ (Higgins and Altman, 2008) and interpreted as “might not be important” (0–40%), “may represent moderate heterogeneity” (30–60%), “may represent substantial heterogeneity” (50–90%), and “considerable heterogeneity” (75–100%) (Deeks et al., 2019). Sensitivity analyses will be conducted when missing data suggest major bias. Studies with high risk of bias (i.e., allocation and outcome assessment not concealed) will be excluded.

### 2.4. Statistical analysis and data synthesis

One author will enter data into Review Manager 5 (RevMan, 2014). When we consider homogeneity among studies, we will perform a meta-analysis by pooling the appropriate data.

We will consider similar outcomes measures among studies, like type of neurological disease, relationship with the patient, type of intervention, and telerehabilitation application.

## 3. Discussion

Rehabilitation processes need to focus on the health of caregivers, who interpret caring as a duty and spend time and energy with others. Informal caregivers of patients with chronic diseases, especially neurological disorders, have intense physical and mental overload and may become ill due to stress, anxiety, and burden.

Telerehabilitation has been used as a strategy for patients with motor and neurological disorders and their caregivers. It can also be used for people who cannot leave the house due to lack of time, physical or mental health, or financial conditions. Besides, this approach has already been approved, due to comfort when using the tool, improvements in patient care, and in the caregiver's quality of life Chi and Demiris (2015). This review will summarize the impacts of telerehabilitation on the health of caregivers of patients with neurological disorders and subsidize the need for attention to their quality of life.

Most caregivers report difficulties in directing their time to their own activities, prioritizing patient care, and reducing their time for self-care (Son et al., 2007; Schulz and Sherwood, 2008). Thus, often physical illness, such as joint and muscle pain, or mental illness, such as stress and depression.

From our findings, telerehabilitation strategies for public health to systematize methods in order to improve the care for caregivers, identifying the better way to manage the assistance, use, aims, and outcomes. We will also highlight the need to focus on the health of caregivers of patients with neurological disorders.

### 3.1. Strengths and pitfalls

A field-specific structure was established to guide each phase throughout the review, in order to obtain information for health professionals about the characteristics of care for the caregiver, who suffers from the daily burden of the disease with housework and leaves their health in the background, not prioritizing his own problems and duties.

The review may allow the analysis of methodologies and interventions used with caregivers, evaluating their applicability of telerehabilitation in the caregiver's health.

The possible scarcity of studies that primarily focus on caregiver rehabilitation strategies and/or that carry out assessment and intervention in this population may be a limiting factor for the review.

## Ethics statement

Ethical review and approval was not required for the study on human participants in accordance with the local legislation and institutional requirements. Written informed consent for participation was not required for this study in accordance with the national legislation and the institutional requirements.

## Author contributions

ES conceptualized the protocol and established eligibility criteria, data extraction framework, and wrote this manuscript. AS, LH, and LM have written this protocol. TR has elaborated on the strategy search. AL research project organization, manuscript review, and critique. RV manuscript review and critique. All authors made substantial contributions to this protocol, read, and approved the final version.

## Funding

This study was partially funded by the Coordenação de Aperfeiçoamento de Pessoal de Nível Superior (CAPES) - Finance Code 001, National Council for Scientific and Technological Development and Ministry of Health, through a Decentralized Execution Term (TED 132/2018). The authors are also grateful to the Federal University of Rio Grande do Norte and the Brazilian Ministry of Education (MEC).

## Conflict of interest

The authors declare that the research was conducted in the absence of any commercial or financial relationships that could be construed as a potential conflict of interest.

## Publisher's note

All claims expressed in this article are solely those of the authors and do not necessarily represent those of their affiliated organizations, or those of the publisher, the editors and the reviewers. Any product that may be evaluated in this article, or claim that may be made by its manufacturer, is not guaranteed or endorsed by the publisher.
