# Supplementary material for: Impact of physiotherapy with telerehabilitation on caregivers of patients with neurological disorders: A systematic review protocol
Source: Front Aging Neurosci. 2022 Sep 2;14:951397. doi: 10.3389/fnagi.2022.951397 (PMC9483827; doi:10.3389/fnagi.2022.951397)
Supplement: Supplementary file 1 [file Data_Sheet_1.pdf]

Appendix 1

Search strategy

Terms for participants:

caregiver* or care giver* or caregiving* or carer* or assistant* or famil* carer* or famil* support* or famil* care or famil* caregiver* or famil* care giver* or famil* caregiving* or famil* assistant*

Terms for interventions:

tele or digital or internet-based or web-based or home or remote or virtual or *health or electronic health or mobile health

AND

physiotherapy or physical or exercise or training or rehabilitation or rehab or therapy or fitness or activity or treatment or intervention or mobility

Terms for study types:

trial or clinical trial or control* trial or control* clinical trial or random* control* trial or random* trial or random* clinical trial or random* sampl* or random* or experimental* or experimental* design* or experimental study or experimental research or placebo or groups

MEDLINE search strategy

TX (caregiver* or care giver* or caregiving* or carer* or assistant* or famil* carer* or famil* support* or famil* care or famil* caregiver* or famil* care giver* or famil* caregiving* or famil* assistant*) AND SU ((tele or digital or internet-based or web-based or home or remote or virtual or *health or electronic health or mobile health) AND AB (physiotherapy or physical or exercise or training or rehabilitation or rehab or therapy or fitness or activity or treatment or intervention or mobility)) AND PT (trial or clinical trial or control* trial or control* clinical trial or random* control* trial or random* trial or random* clinical trial or random* sampl* or random* or experimental* or experimental* design* or experimental study or experimental research or placebo or groups)
