# Supplementary material for: Impact of physiotherapy with telerehabilitation on caregivers of patients with neurological disorders: A systematic review protocol
Source: Front Aging Neurosci. 2022 Sep 2;14:951397. doi: 10.3389/fnagi.2022.951397 (PMC9483827; doi:10.3389/fnagi.2022.951397)
Supplement: Supplementary file 2 [file Data_Sheet_2.pdf]

Appendix 2

Table for data extraction

Study design	Author(s) and year of publication	Location	Clinical diagnosis of people receiving care	Characteristics of caregivers	Assessment	Intervention	Results
